# Impulsivity: A Dimensional Perspective in PD and ED. Comparison of Results in a Case-Control Study.

**DOI:** 10.1192/j.eurpsy.2024.976

**Published:** 2024-08-27

**Authors:** M. Pérez-Lombardo, Í. Alberdi-Páramo, W. Ayad-Ahmed, J. L. Carrasco-Perera, M. Díaz-Marsá

**Affiliations:** ^1^Psiquiatría, Hospital Clínico San Carlos; ^2^Psiquiatría y Medicina Legal, Universidad Complutense de Madrid, Madrid, Spain

## Abstract

**Introduction:**

Personality Disorders (PD) - specifically Borderline Personality Disorder (BPD), and certain Eating Disorders (ED) share common clinical features. One of these features is impulsivity, studied individually in each diagnostic group, and scarcely used to encompass specific profiles of these patients.

Understanding the common clinical variables of this patient population would facilitate therapeutic efforts and enable greater precision regarding the prognosis of these patients.

**Objectives:**

This study aims to study impulsivity in a group collectively formed by BPD and ED, compared to a control group, in contrast to the individualized study approach typically conducted in the literature.

**Methods:**

A cross-sectional descriptive study is conducted to assess impulsivity as a common diagnostic variable in a group of PD and ED in comparison with a healthy control group. The sample was collected between 2016 and 2019 at the Hospital Clínico San Carlos, totaling 108 subjects.

**Results:**

A statistically significant difference is observed (p<0.005 in all scales) in total impulsivity, cognitive impulsivity, motor impulsivity, and unplanned impulsivity in the cases group comprising patients diagnosed with PD and ED, compared to the control group from the general population.

**Conclusions:**

Impulsivity is closely related to the concept of borderline personality disorder. This analysis also includes eating disorders, with the difference from the control group still statistically significant.

The presence of common clinical variables in these groups (PD and ED) may have clinical and therapeutic implications that differ from those pursued thus far. This allows moving away from the categorical model and understanding these disorders from a more enriching and advanced dimensional perspective.”

**Disclosure of Interest:**

None Declared

